# The three most important things about origins: location, location, location

**DOI:** 10.1002/msb.145202

**Published:** 2014-04-04

**Authors:** Nicholas Rhind

**Affiliations:** ^1^ Biochemistry and Molecular PharmacologyUniversity of Massachusetts Medical SchoolWorcesterMAUSA

## Abstract

The reasons why some DNA replication origins fire earlier than others have remained elusive. New work by Gindin *et al* suggests that the distribution of replication origins, not their timing *per se*, is the major determinant of the timing of genome replication in human cells.

In any eukaryotic genome, some regions replicate early, while others replicate late (Rhind & Gilbert, 2013). In yeast, this observation applies at the level of individual origins and in metazoans—where the lower spatial resolution of replication timing profiles does not allow the identification of individual origins—it applies to replication domains, large regions (100 kb to 1 Mb) containing many origins. In both cases, it is believed that replication timing is determined by the timing of origin firing, and it has been widely assumed that there exist mechanisms regulating the times at which different origins fire.

Gindin *et al* (2014) take a systems approach to analyze replication timing in the human genome. They build a simple model of replication kinetics that considers two inputs to simulate the genome‐wide profile of origin firing. The first input is the “initiation probability landscape” (IPLS), the probability that replication will initiate at any particular site in the genome. In a genome with well‐defined origins, such as budding yeast, the IPLS‐specified probability of origin firing would be high at efficient origins, low at inefficient origins, and zero everywhere else. In mammalian genomes, where replication initiation events have been proposed to be distributed over broad zones (Hamlin *et al*, 2008), the IPLS probability would be distributed across such zones. The second input is the abundance of a rate‐limiting activator, competition for which regulates the firing of origins. Thus, the chance of replication initiating at a particular site is simply the product of the probability from the IPLS and the number of available activators. The inclusion of such a rate‐limiting activator in the model is in agreement with experimental studies suggesting that rate‐limiting activators regulate replication kinetics in yeast and theoretical studies arguing that such activators would produce observed replication kinetics (Goldar *et al*, 2008; Patel *et al*, 2008; Gauthier & Bechhoefer, 2009; Mantiero *et al*, 2011). The rate‐limiting activators in budding yeast are the Dbf4‐dependent kinase (DDK) replication kinase and various DDK and cyclin‐dependent kinase (CDK) substrates (Mantiero *et al*, 2011), but other factors may be limiting in other species. In their model, Gindin *et al* use a generic replication fork factor. Given these two inputs, the model simulates replication in millions of cells and then averages the results to produce a replication timing profile.

Various genomic features including GC content, gene density and histone modifications were used to create IPLSs, the idea being that the features that produce simulated replication profiles similar to those experimentally observed would be the most likely determinants of origin timing. Of the 173 features tested, an IPLS based on DNase I hypersensitivity produced the most realistic replication timing profiles, which matched the experimental profiles well (correlation of *r* = 0.86). Moreover, DNase I hypersensitivity profiles from specific cell lines recapitulated the differences in timing profiles between these cells. This result is reminiscent of the observation that replication timing is highly correlated with the 3D structure of the genome (Ryba *et al*, 2010). Both of these features—DNase accessibility and 3D conformation—integrate aspects of DNA sequence and chromatin modification and thus presumably report on the elements of genome structure with the largest influence on origin activity better than any other single genome feature. In any case, this remarkably simple result suggests that the distribution of DNase I hypersensitive sites accurately predicts the distribution of replication origins in the human genome.

Even more remarkable is the fact that the IPLS created from the location of DNase I hypersensitive sites contains no explicit timing information. The model accurately predicts replication timing even if origins in late‐replicating parts of the genome are assigned the same firing probability as origins in the early‐replicating parts. The key to understanding this counterintuitive result is realizing that timing information in the IPLS does not come from the timing of individual origins, but instead from the distribution of the origins and, in particular, from the fact that there are many more DNase I hypersensitive sites in early‐replicating parts of the genome (Fig 1A). Therefore, even though an origin in a late‐replicating part of the genome is just as likely to fire as an origin in an early‐replicating part, there are so many more potential origins in the early‐replicating regions that the rate‐limiting activator rarely finds origins in late‐replicating parts of the genome and thus they rarely fire. This situation changes, of course, once the early‐replicating parts of the genome have replicated. At that point, the origins in the late‐replicating parts of the genome have no more competition for the rate‐limiting activator, and they are able to initiate efficiently. It is worth noting that the rate‐limiting activator, which allows the rare origins in late‐replicating parts of the genome to fire efficiently in late S‐phase, is a crucial aspect of the model. Simply using DNase I sites to predict origin location in a model that does not include competition for a rate‐limiting activator does not produce realistic replication timing profiles.

**Figure 1:**
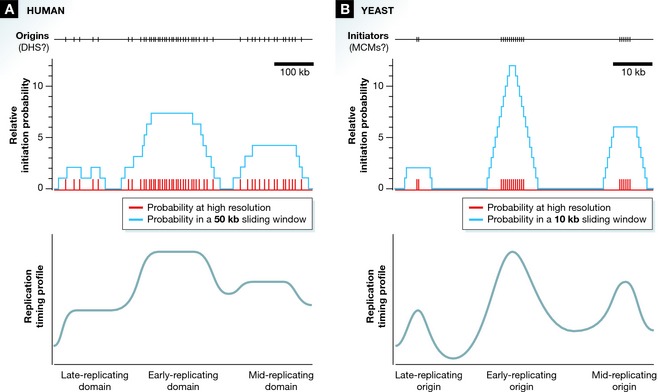
The effect of origin location on replication timing (A) The distribution of origins (which correlate with DNase I hypersensitive sites) across a region of a human chromosome is shown on top. The middle graph shows the probability of initiation at each origin, defined by Gindin *et al* as the “initiation probability landscape” (IPLS). In red, the firing probability at high resolution indicates that the probability is equal at all origins. In blue, the probability in a 50‐kb sliding window shows that a higher density of origins in early‐replicating domains leads to a much higher cumulative probability of origins firing in such domains. (B) A similar scenario has been proposed for yeast, albeit at higher resolution. The top line shows the distribution of hypothetical initiators (suggested to be MCM complexes) loaded at origins. As in (A), the cumulative probability (in a 10‐kb sliding window) of multiple initiators at early origins makes initiation at such origins more likely.

The concept that origin location and not the timing of individual origin firing determines replication timing fits well with a developing framework for understanding replication kinetics. The gist of that framework is that replication timing is a systemic phenomenon that emerges from the stochastic firing of replication origins (Bechhoefer & Rhind, 2012). A recent model for replication timing in budding yeast also posits that replication timing is not regulated by the firing times of individual initiators, but rather by the distribution of initiators across the genome (Yang *et al*, 2010). In the yeast model, however, different numbers of initiators are proposed to be bound at individual origins. Thus, the yeast model parallels the IPLS model, but at higher resolution. Instead of having more origins at early‐replicating domains and fewer at late‐replicating domains, it proposes more initiators bound at early‐firing origins and fewer at late‐firing ones (Fig 1B). The hypothetical initiators in yeast have been proposed to be the MCM complex, but this remains to be confirmed experimentally. Nevertheless, a key factor in both models is spatial resolution: There are so many potential initiation sites that, at the available experimental resolution, individual initiation events cannot be discerned and all that is visible is the average replication profile. A rigorous test of these models will come from high‐resolution single molecule analysis of replication kinetics, which will be able to deconvolve the heterogeneous nature of replication kinetics and directly determine where and when replication initiates.

## Conflict of interest

The author declares that he has no conflict of interest.
